# Designing Efficient Flash-Calcined Sediment-Based Ecobinders

**DOI:** 10.3390/ma15207107

**Published:** 2022-10-13

**Authors:** Mouhamadou Amar, Mahfoud Benzerzour, Nor-Edine Abriak

**Affiliations:** 1 IMT Nord Europe, Institut Mines-Télécom, Centre for Materials and Processes, F-59000 Lille, France; 2 Univ. Lille, Institut Mines-Télécom, Univ. Artois, Junia, ULR 4515—LGCgE—Laboratoire de Génie Civil et Géo-Environnement, F-59000 Lille, France

**Keywords:** sediment, characterization, flash calcination, substitution, pozzolanic activity

## Abstract

To ensure the optimum navigation of boats and protection against flooding, waterways and ports are regularly dredged. The volume of dredged materials represents 56 million m^3^ in France and 300 million m^3^ in Europe. These materials show a high potential for a use as supplementary cementitious material (SCM). In this paper, sediments treated by the flash calcination method (STFC), which is based on a low-energy consumption process, are utilized as a mineral admixture in a cementitious matrix. The results of the physical, chemical, and mineralogical characterization prove that this heat treatment has an interesting impact on the final properties of the sediments. Mortars based on the flash-calcined product have comparable mechanical properties to control mortar. For a substitution rate below 10%, the performances are even equivalent to a metakaolin (MK80)-based mortar. Calorimetry testing demonstrated that calcined materials also improve hydration processes in the cement matrixes by generating additional heat release due to sediment pozzolanic activity. Across this study, it is shown that waste material including sediment can be transformed after optimized heat treatment into a valuable resource for the building and infrastructure sector.

## 1. Introduction

In the early 21st century, the environment has become one of the biggest issues. Climate change and environmental degradation pose an existential threat to Europe (EU) and the rest of the world. To implement a new growth strategy regarding environmental issues, several countries, notably in the EU, are conducting the “Green Deal” approach leading to sustainable development. The principle is to dissociate economic growth from resource use and also to reach zero greenhouse gas emissions by 2050. Recently, the International Conference on Climate Change (COP 26, 2022) was held in Scotland and endeavored to regulate the global carbon market, climate change, worldwide temperature rise, water supply problems, etc. Several government policies nowadays aim to optimize productivity through better efficiency in order to reduce the use of natural resources, energy consumption, and waste production.

Ordinary Portland Cement (OPC), which is the essential component in concretes and mortars, is responsible for at least 7% of global CO_2_ emissions resulting from the annual worldwide manufacture of 4.6 billion tons of this product [1]. According to recent predictions, the share of global CO_2_ emissions attributed to cement production is likely to reach 25% in 2050, hence the urgent need to improve its sustainability [2]. In China, the cement industry, which is one of the most energy-intensive sectors emitting 1.2 to 2 billion tons of carbon per year [3], is developing technologies to reduce its impact. Through Carbon Capture and Storage (CCS), alternative raw material use, and energy efficiency, it is targeted to cut down 30% CO_2_ emissions by 2050 [4,5]. The adopted strategy in Europe and the USA is also similar through the use of Limestone Calcined Clay Cement (LC3) and nanotechnologies [1,6].

In the construction sector, the aggregate need was 445 million tons in France in 2021. More than 96% of these aggregates are of natural origin. In 2019, the turnover in the aggregates sector increased by up to 3.66 billion euros and the ready-mixed concrete (RMC) market production was 3.77 billion euros [7]. Meanwhile, cement consumption in France was approximately 18 million tons. This constitutes huge financial profits for industrial companies but is also alarming for the environment and resource use. For all these reasons, sediments can potentially be used as suitable materials (as SCMs) in the construction sector [8,9,10,11,12]. The clinker content can be reduced significantly by replacing cement with SCMs, which have multiple effects: pozzolanic, hydraulic, and filler properties. Cement’s high level of pollution is mainly due to limestone decarbonization, which is fundamental in order to achieve the correct chemical chemistry of Portland cement [13].

Saving natural resources is crucial, and environmental regulations are increasing. It is therefore sensible to develop the use of alternative materials in the construction sector. Several projects (ECOSED chair, FISP (US), USAR (EU), SETARMS, SURICATES, etc.) have already studied and considered using sediments as a resource for sustainable development purposes [8,9,10,11,12,14]. Every year in France, around 56 million m^3^ [15] of sediments are dredged, stored, and treated. In Europe, the total volume is 300 million m^3^, while in Brazil, it has reached 80.3 million m^3^ [16,17]. Due to the high and growing demand for cement, the availability of conventional SCMs, such as blast furnace slag, limestone filler, silica fume, and natural pozzolans will not be enough to fully cover it. However, the beneficial use of dredged material relies strongly on numerous factors: mineral composition, grain size distribution, and physicochemical characteristics [18]. Furthermore, for this purpose, numerous scientific obstacles need to be resolved, including the inorganic contaminant content of sediments, such as heavy metals (lead, copper, chromium, etc.), salts, cyanides, and organic hydrocarbons (PAHs, PCBs, and TBT) [19].

Transforming raw sediment, considered waste, into a new material resource still faces technical challenges. One of the options is to process the sediments into an SCM by calcination, leading to the activation of mineral fractions. Heat treatment through “direct” or traditional calcination using a laboratory kiln has already been studied in sediments to improve some properties [20,21,22,23,24,25,26,27,28], but this method is expensive with high energy consumption and environmental impact. However, another innovative technique, called “flash calcination”, has been applied in some studies for the activation of clays and to transform kaolin into metakaolin [29,30,31,32,33,34]. Cremona et al. [35] investigated the properties of fresh and hardened concrete and the durability of several metakaolin-based binders. It was concluded that flash-calcined clays improve workability, porosity, and creeping. Similar conclusions were stated by Sullivan et al. [36], showing that MK contributes to prolonging the service life of infrastructures. Danilevich et al. [37] activated thermally aluminum hydroxide in gibbsite using a reactor called TSEFLAR. Examining the characteristics of the final product, the study has shown that process parameters such as temperature, the residence time of the particles, and raw material consumption play a major role in activation processes. This new calcination technique has the advantage of lowering energy consumption and gas release due to the quickness of the process. As a matter of fact, this process includes dehydroxylation that activates the material with thermal excitation between 700 °C and 850 °C. For products containing undesirable non-active materials, complex calcium-based minerals, sulfur-rich phases [38], or phosphates [39], flash calcination can be considered to be more advantageous than traditional treatment methods such as rotary calcination. Flash calcination is also able to activate Muscovite, illite, phlogopite, and kaolinite, which are among the clay minerals that show pozzolanic activity after appropriate heat treatment and are generally present in sediment minerals. Such results were previously obtained by Ambroise [40] after calcination at 750 °C. This triggers and activates amorphous phases [30,41]. The activation of the clay fractions present in sediments has recently been studied [2,26,42,43,44]. The final product is also usable as SCM for traditional Portland cement replacement [45] and in geopolymer applications [46]. Amar et al. [47] studied the reactivity of flash-calcined sediment. By using various pozzolanic tests, it was demonstrated that the calcination process has a positive impact on the pozzolanic activity of sediments [48]. Snellings et al. [26,42] also used the flash calcination technique on the same sediment studied by Van Bunderen et al. [25]. This last study investigated the influence of the temperature of treatment and the final reactivity of sediment. The authors tested three calcination temperatures: 820 °C, 865 °C, 905 °C, and showed the influence on the final product properties. Van Bunderen et al. [34] also studied the effect of flash-calcined sediment on mechanical properties and the shrinkage of concretes. After testing cement replacement at 20, 30, and 40 wt%, the results clearly demonstrated that flash-calcined sediments are suitable for the production of sustainable cement and concrete. Inocente et al. [33] recently used this activation technique to produce highly reactive metakaolin. The chosen calcination temperatures ranged from 900 °C to 1100 °C. It was also shown that flash calcination is suitable for transforming unreactive material into a product with a high degree of amorphization.

The flash calcination treatment technique based on a low-energy process is studied in the following. This innovative method is not widely used in the waste-reuse sector. This method was shown to be capable of enhancing the pozzolanic activity of sediments. As a result, these materials that were initially considered waste can be transformed into a suitable SCM for the construction sector.

First, this paper will focus on the characteristics of sediments treated with the flash calcination method (STFC) and will then highlight the impact of the treatment. Next, mortars using STCF as eco-efficient SCM will be formulated and tested. Finally, a comparative study will be made of raw sediment (RS), metakaolin (MK), and limestone filler (LF).

## 2. Materials, Methods, and Experimental Work

### 2.1. Materials

Portland cement CEM I 52.5N (OPC), which complies with European standard EN 197-1 (2012), was used in this study. The mortar formulations were based on a siliceous standardized river-origin sand (ISO 679: 2009), with rounded grains.

The marine sediments were dredged from the Grand Port Maritime de Dunkerque (GPMD), situated in the North of France. Using an oven at 40 °C, all raw sediments were dried, then crushed, and sieved at 120 µm. In this study, the flash-treated sediments are designated by STFC, RS = raw sediment, NS = natural sand. MK80 is a metakaolin with Dmax = 80 µm, and LF80 is a limestone filler with Dmax = 80 µm.

### 2.2. Characterization Techniques

Different physical and chemical methods were used to identify the characteristics of the materials. Granulometry analysis was performed using a laser diffraction particle size analyzer COULTER LS12330. With this device equipped with a dry module, the particle distribution was determined from 0 to 2 mm. The BET (Brunauer–Emmett–Teller) tests, also called mass area and which are an estimation of fineness (NF EN ISO 18757 [49]), were performed on a Micromeritics ASAP2020 analyzer using nitrogen gas. The samples were outgassed for 16 h at 40 °C. Blaine surface was measured following the NF EN 196-6 [50] standard. The organic fraction (LOI) was also determined (XP P94-047 [51]), and the total organic carbon (TOC) content was evaluated. The specific density of the products was determined by a MICROMETRICS AccuPyc1330 helium pycnometer (NF EN 1097-7 [52]). Heat release investigations using a semi-adiabatic calorimeter CERILH (Langavant method) according to the NF EN 196-9 [50] standard were also carried out for the first seven days following initial cement hydration. The apparatus was 400 × 160 mm with a thermally sealed container equipped with heat sensors. Differential Scanning Calorimetry (DSC) and Thermogravimetric Analysis (TGA) were performed on a NETZSCH STA 449 using nitrogen gas. The analysis program was set to work between 40 °C and 1100 °C and CO, CO_2,_ and water detection were activated. The mineral composition was investigated using X-ray diffraction on a Bruker D8 device equipped with a Co anticathode. The Rietveld refinement method was also applied, especially for the quantitative measurements of the clay fractions. Analysis was conducted as follows: between 6–80° angle 2θ, 0.2° pitch, and acquisition time 0.5 s. Scanning Electron Microscope (SEM) analysis was performed using a Hitachi S-4300SE/N with a field emission gun; the speed voltage was adjusted to 5 kV.

### 2.3. Mortar Preparation

The composition of mortars used to study mechanical strength is given in Table 1. The consistency test was executed according to NF EN 196-3:(2009). This test guarantees to have an equivalent fresh state between the control mortar and those including SCMs to keep results comparable. Mechanical strength was evaluated using prismatic mortar specimens 4 cm × 4 cm × 16 cm (NF EN 196-1 [53]). RM is the control mortar. MRS and MSTFC are the respective raw-sediment-based and flash-calcined-based mortars. Formulations using MK and LF are designated by MMK80 and MLF80 in this order.

### 2.4. Flash Calcination Method

**Figure 1:**
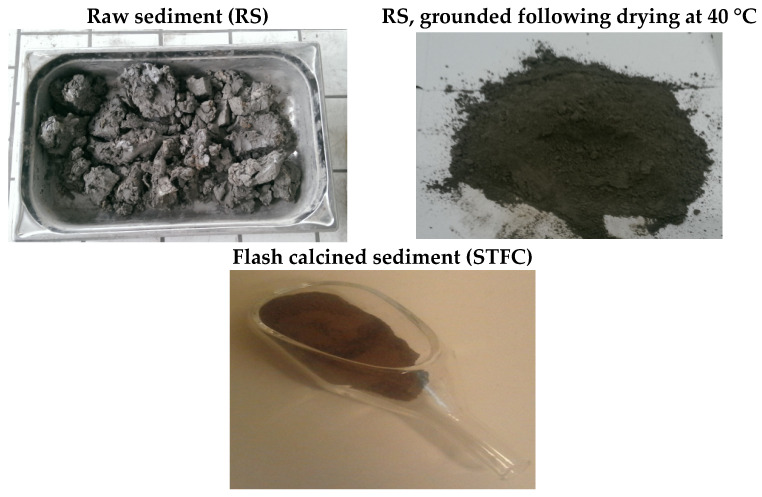
The visual aspect of sediment through treatment stages.

Flash calcination is a heat treatment technique consisting of the exposure of finely ground products to high temperatures in the presence of air. In flash calcination, gas–solid heat transfer is exploited effectively to calcinate the material quickly. This technique was developed in France by Salvador in 1992 [31]. Originally, the flash calcination technique was applied to activate clays such as kaolinite and to trigger their pozzolanic properties. This treatment method provokes a dihydroxylation process following dehydration [26,30,54]. This fits with the elimination of a hydroxyl bond (−OH) that occurs between 450 °C and 750 °C [55]. In addition, as the flash process is spontaneous, the flash calcination initiates amorphous phases and therefore boosts the reactivity of the product [2,56]. In fact, the temperature rise can be estimated from 0.5 to 1.5 × 10^4^ °C/s [57]. Due to the rapid process, the material obtained has a lower density and higher porosity translating to increased reactivity. Direct calcination using a laboratory kiln at 750 °C for a residence time between 1 h to 3 h can lead to such results. However, in this case, energy costs and the CO_2_ footprint, in particular, are usually high. In flash calcination, the product is heated rapidly (up to 1200 °C), held at this high temperature for a short period (0.1 to 1 s), and finally quenched rapidly with the counter flow of cold air before storing [44,54]. The visual aspect of the sediment throughout the treatment process is also shown in Figure 1.

**Figure 2:**
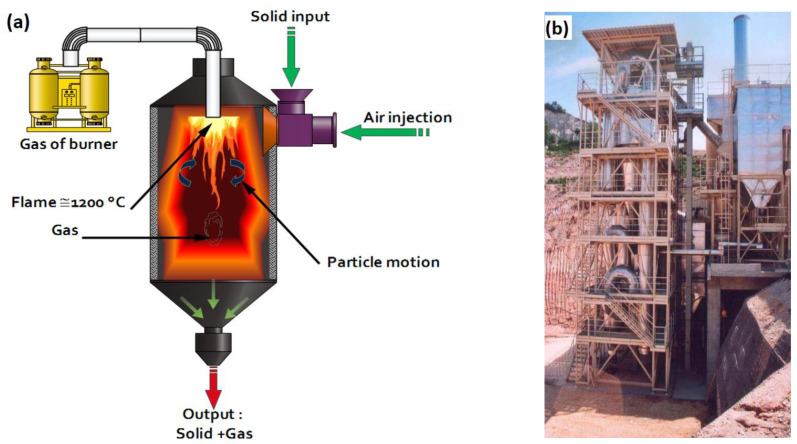

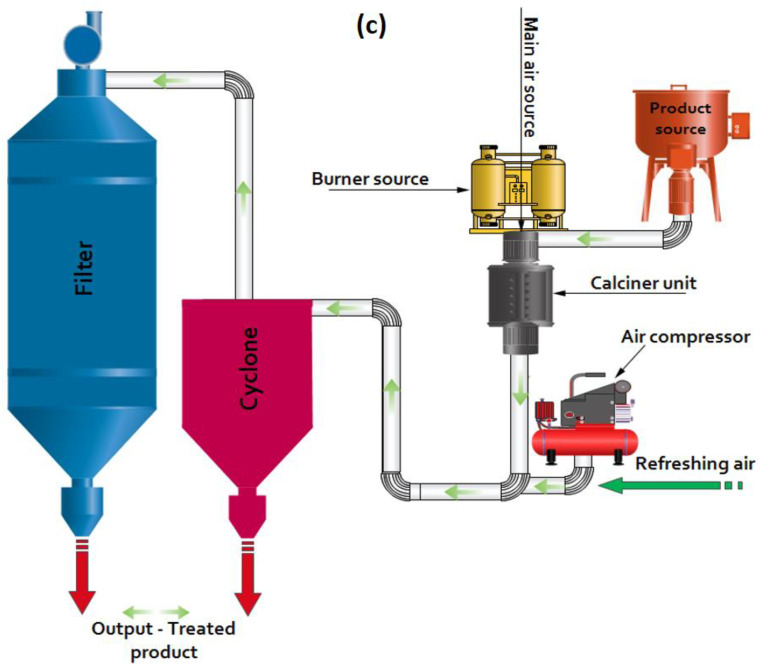
(**a**) Flash calciner chamber; (**b**) Semi-mobile flash tower [22]; (**c**) Pilot installation.

Adjusting the flash calcination process requires the control of two parameters: exposure time and temperature, which can reach 1200 °C inside the calciner unit [30]. The energy used in this flash calcination process can be evaluated at 2 GJ/t according to San Nicolas [30,58]. This study aimed to apply this treatment process to the marine dredged sediments, which will be adapted to activate sediment phases (including clays). The calcining chamber unit, flash tower, and pilot installation are shown in Figure 2.

## 3. Experimental Results and Discussion

### 3.1. Physical Characterization

#### 3.1.1. Granulometry

**Figure 3:**
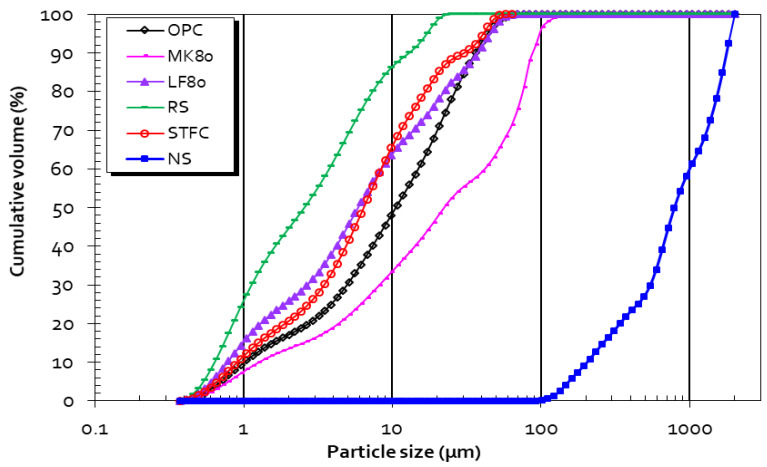
Particle size distribution for OPC, MK80, LF80, NS, RS, and STFC.

The results of the laser granulometry test are shown in Figure 3. The calcination process affects the particle size distribution (D_50_ = 2.46 µm for RS, and D_50_ = 5.93 µm for STFC). The sintering effect between particles may be the main reason RS is finer than STCF and Portland cement. This result agrees with SEM observations showing the presence of very fine particles in the RS sample. Particle size distribution plays a major role in pore size distribution. It was also shown through fractal analysis that it impacts hydration, porosity, durability, and mechanical resistance [59,60].

#### 3.1.2. Physical Properties

The results of the BET and the Blaine tests are given in Table 2. The results demonstrate that the flash calcination process has an impact on the fineness of the materials. This effect is the outcome of the organic matter elimination, which wraps the sediment particles. Therefore, with the calcination, organic matter is eliminated, and the BET/Blaine value decreases. Fineness is a parameter connected to the reactivity of the material and its performance. For the flash-calcined sediments, the LOI (1.7%) of the final product is low and proves the efficiency of calcination.

**Figure 4:**
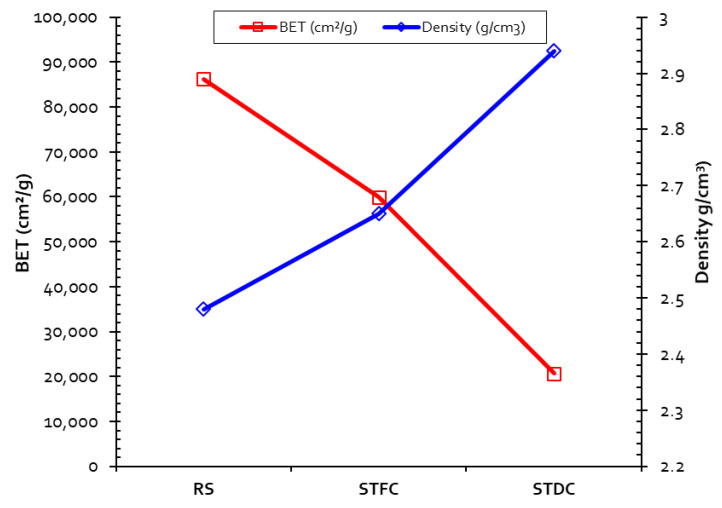
Evolution of density and BET surface area for RS, STFC, and STDC.

Generally, a decrease in the specific surface area is observed after the consistent thermal process. This variation is inversely proportional to density, as shown in Figure 4. It may indeed be explained by physical and chemical modifications due to sintering [62]. In Figure 1, STDC is the sediment treated by the traditional calcination process. Snellings et al. [42] measured the effect of the flash calcination process on sediment and established that the higher the calcination temperature, the lower the specific surface area. According to [29], the calcination process induces the formation of spherical particles that are the product of multiple melting–sintering and destructuration processes.

Finally, it is important to highlight that a heat treatment usually produces the following effects [63]: (a) Modification of the bulk density (increase and decrease from 600 °C); (b) Modification of the specific area (increase and decrease from 400 °C); (c) Decrease of internal porosity by densification; (d) Change in grain size (D_50_ increases strongly at 700 °C).

#### 3.1.3. TGA and DSC Results

**Figure 5:**
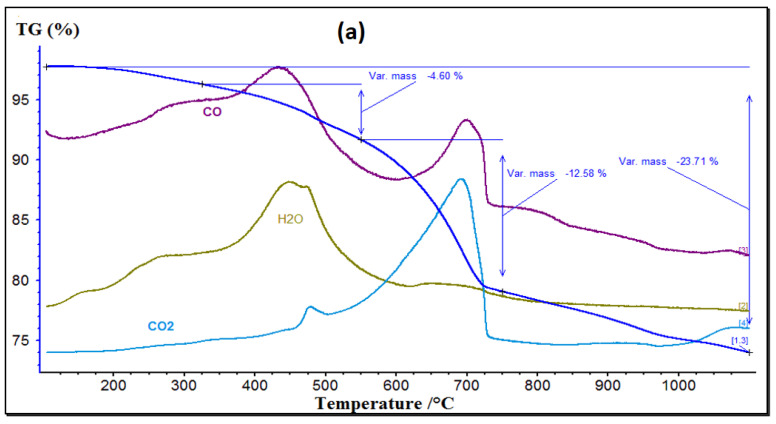

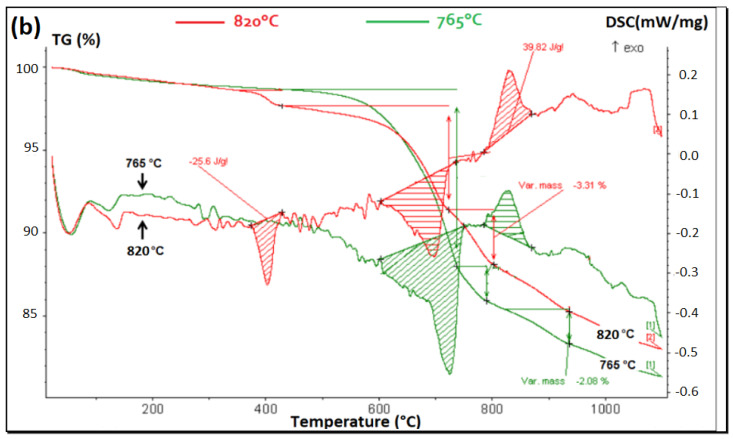
(**a**) TGA results for RS and (**b**) TGA on pre-flash-calcined sediment (STFC).

The TGA results displayed in Figure 5a highlight a peak in the mass loss at 450 °C corresponding to the dehydration phase. The release of carbon monoxide (CO) and carbon dioxide (CO_2_) at 420 °C and 480 °C, respectively, is due to the combustion reactions of organic matter and organic pollutants (PAHs, PCBs, TBTs) present in the sediment [64]. A second CO_2_ peak appearing at 700 °C is caused by the decarbonation of calcite and dolomite [23]. These calcareous materials originate from the shells of marine species naturally present in sediments. According to these results, the temperature that guarantees organic matter elimination and also complete decarbonation must be greater than 700 °C.

The choice of the optimal calcination temperature relies on the DSC results depicted in Figure 5b. This test was performed on sediments that were flash calcined at 765 °C and 820 °C. The first peak at 400 °C probably corresponds to the organic matter decomposition as stated in Figure 5a. The analysis reveals the recrystallization area at 810 °C is narrow, indicating that recrystallization was incomplete. These results prove that flash calcination at 765 °C or 820 °C leads to similar results, but is more energy costly at 820 °C. Due to its efficiency, the choice was made to adopt 765 °C as the optimized temperature of calcination.

### 3.2. Chemical and Mineralogical Analyses

The purpose of the chemical analysis was to determine the proportion of each chemical element using X-ray Fluorescence (XRF) analysis (see Table 3). These values are close to those identified in previous studies [65,66] conducted on GPMD sediments. As for the calcined sediments, the main oxide constituents SiO_2_, Al_2_O_3_, and Fe_2_O_3_ were similar to the RS values. These results confirm the possible moderate impact of the calcination process on chemical composition. The composition of cement is shown in Table 4 and corresponds to commercial Portland cement regarding its chemical composition.

### 3.3. X-ray Diffraction Analysis (XRD)

**Figure 6:**
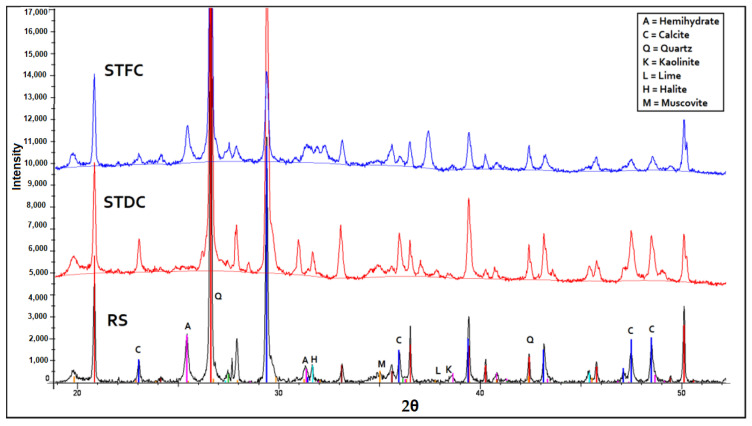
XRD analysis of RS, STDC, and STFC.

This test revealed the high presence of quartz (SiO_2_), calcite (CaCO_3_), and other phases such as pyrite (FeS_2_) or clays (kaolinite) in the RS. The results of the XRD analysis performed on the RS, STDC (traditional calcination) and STFC materials are given in Figure 6 and show major crystalline modifications. The heat probably initiated some physicochemical processes. The XRD clearly demonstrates a drop in the occurrence of crystalline phases such as calcite due to the decarbonization phase. Clay phases such as kaolinite must be transformed into reactive metakaolin [30,56]. It is also noted that there was an initiation of phases such as anhydrite (CaSO_4_), which is the result of reactions between calcium carbonates and sulphate. Anhydrite can modify cement hydration reactions as stated in the investigation led by Snellings et al. [42]. Furthermore, mineral additions with a high calcite (CaCO_3_) content must favor the hydration of alite (C_3_S) [67,68]. This effect is particularly accentuated as the calcite content and granularity are relatively high. It seems that in the presence of water, calcium carbonate reacts with alite (C_3_S) and aluminate (C_4_AH_13_) to form hydrated mono-carboaluminate calcium [C_3_A, CaCO_3_, 11H_2_O], which crystallizes in hexagonal platelets [69,70]. 

**Figure 7:**
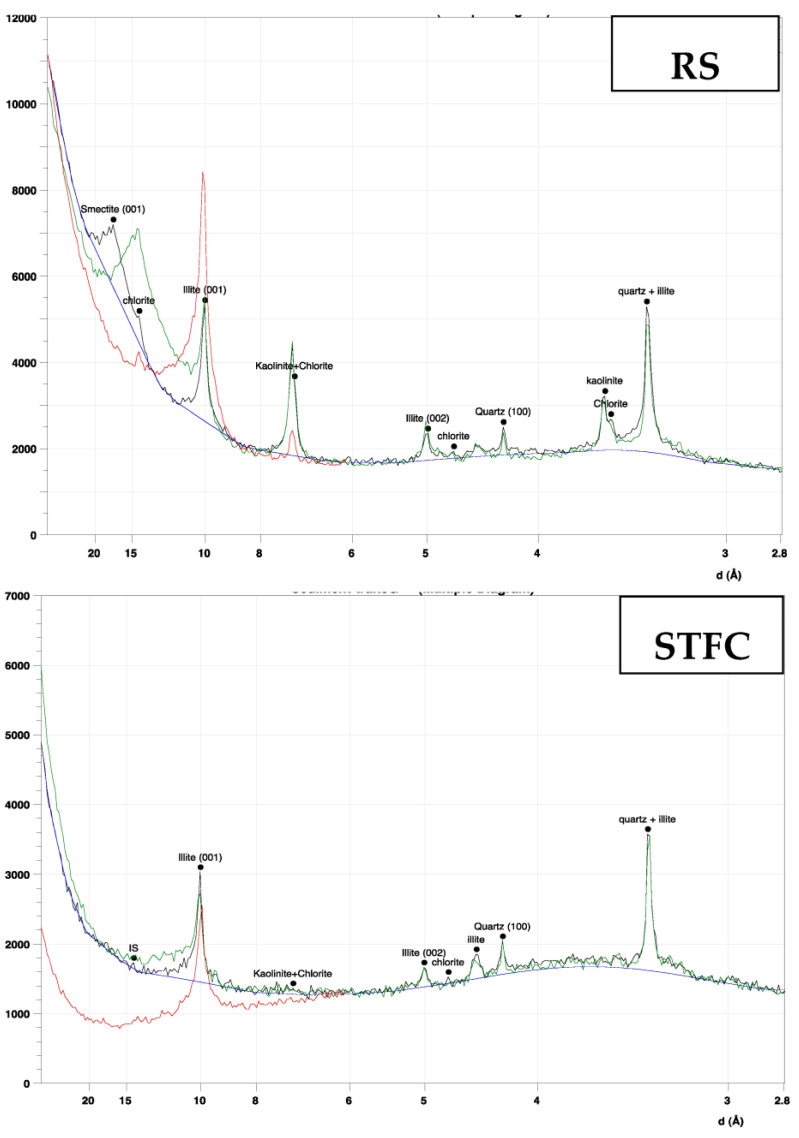
Identification of the mineral clay phases in RS and STFC by XRD.

These analyses were performed on a single-oriented glass slide to identify clay minerals; the results are presented in Figure 7. They are based on the determination of lattice spacings (001) and relative intensities. The calcination process transforms the majority of the clay minerals present into illite (86%). According to Dang et al. [23], the occurrence of newly created phases can be attributed to the heat treatment effect.

### 3.4. XRD Analysis of Clays

The Rietveld semi-quantitative analysis is based on optimization techniques and refinement. The methodology applied relies on the difference between the measured and the calculated diagrams. The statistical criterion generally applied for the fitting quality is the weighted least square error value. The tests were performed on the sediment RS and STCF. Samples were prepared using a single-oriented glass slide to identify clay mineral proportions. This analysis is also based on the determination of lattice spacings (001) and relative intensities. Samples should be prepared carefully, and specific skills are required by the operator. The results are presented in Table 5 and Figure 7.

The analysis reveals that the RS samples are composed of kaolinite, which represents 20% of the clay phases, and chlorite (12%). The main phases are illite (36%) and smectite (32%). The STFC sediment is composed mainly of 86% illite and the remaining 5% of kaolinite. The calcination procedure turns the majority of the clay minerals into illite (86%). These figures demonstrate that heat treatment initiates internal structural modification in clay minerals [42]. This can be partly the cause of chemical activity noticed previously by Amar et al. [47] in these sediments. The occurrence of these newly created phases can be explained by the effect of heat treatment [23,41].

### 3.5. Scanning Electron Microscopy (SEM)

**Figure 8:**
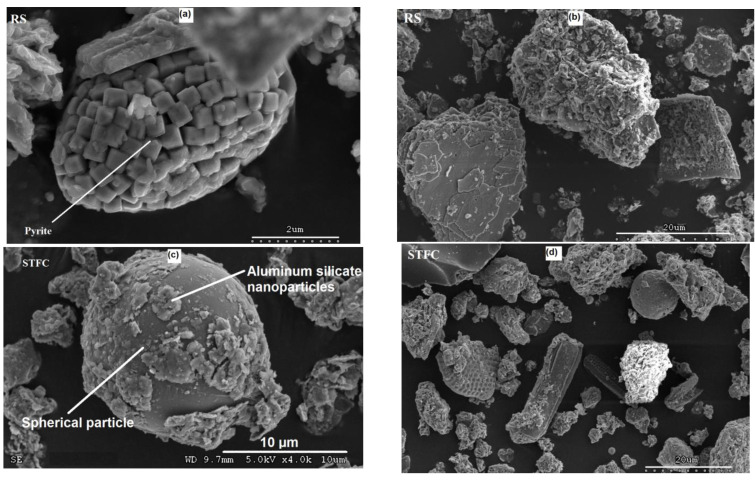
Electron microscopy of RS and treated STFC sediment. (**a**) Pyrite particle, (**b**) plate-like cemented particles, (**c**) Spherical aluminosilicate nanoparticle, (**d**) Organic particles and seashells.

The SEM analysis consists of examining the internal structure of the materials at a very high resolution. SEM analysis provides powerful magnification, up to 500,000 times. As a result, the majority of the micron constituents of mortar can be examined, scrutinized, and studied. In a cementitious matrix, these constituents can be CSH hydrates (≈100 Å) and strips of clay particles (≈10 Å) from SCM, ettringite crystals (≈50 µm), crystals of portlandite Ca(OH)_2_ (≈20 µm), or the morphology of finely-ground material. The results in Figure 8d reveal different particle forms that are mineral or animal (seashell) in origin. These types of particles are commonly observed in sediments. As a result of sedimentation and the presence of minerals such as limestone (CaCO_3_), the phenomenon of cementing between the particles can be observed (Figure 8b). For the RS material, the presence of pyrite particles and some plate-like particles can be clearly distinguished (Figure 8a). Pyrite is a mineral originally present in raw sediments and is visible in Figure 8a. The presence of organic constituents or pollutants that needed to be eliminated justifies the need for an appropriate treatment choice (using heat treatment, for instance).

For STFC, physical changes were confirmed by the SEM observations. The heating process modified the form of the particles with the development of regular spherical particles with a diameter of 20 µm (Figure 8c). In previous studies led by Nicolas et al. [30] and also by Teklay et al. [44], these spherical particles were identified on flash-calcined kaolin clay, representing almost 20% of the final product. Similarly, Claverie et al. [29] showed that these particles originated from an agglomeration process of submicron particles, as shown in Figure 8c. They are mainly composed of gases and nano-particles of aluminum silicates that cover the external surface (Figure 8c). Through later studies, Snellings et al. [26,42] highlighted the local melting process origin of this rounded effect.

### 3.6. Impact of the Presence of Sediments on Cementitious Matrix

Due to their physicochemical composition, sediments modify the cementitious matrix properties [23,41]. In fact, this is materialized by the presence of lime as a result of the thermal conversion of calcite. Depending on their fineness and shape, sediments are able to play a role in filling space and thus contribute to improving overall compactness by reducing the intergranular space. This contributes to enhancing the hydration reactions and ameliorating general properties. However, the presence of constituents resulting from pollution, such as lead (Pb) [71], zinc (Zn) [72], cadmium (Cd), or chromium (Cr) [71], could disturb the normal hydration processes and the setting of mortar [73,74]. The mechanisms that usually lead to the formation of hydrates (CSH) or the setup of the porous network can be disrupted by some of these minerals [71,73,75]. The effect of metal contaminants has been studied by Minocha et al. and Park et al. [71,76]. It has been shown that the presence of inorganic contaminants in the cementitious matrix generally has a detrimental effect on mechanical strength and also on durability properties [76].

The formulated mortars are designated as follows: RM = control mortar (with no SCM); MRS = mortars with raw sediment; MSTFC = mortars including flash-treated sediments; MMK = metakaolin-based mortar, and MLF = mortar with limestone filler. The number after these notations stands for the substitution rate (e.g., MRS5 = 5% cement replacement).

### 3.7. Mechanical Strength

**Figure 9:**
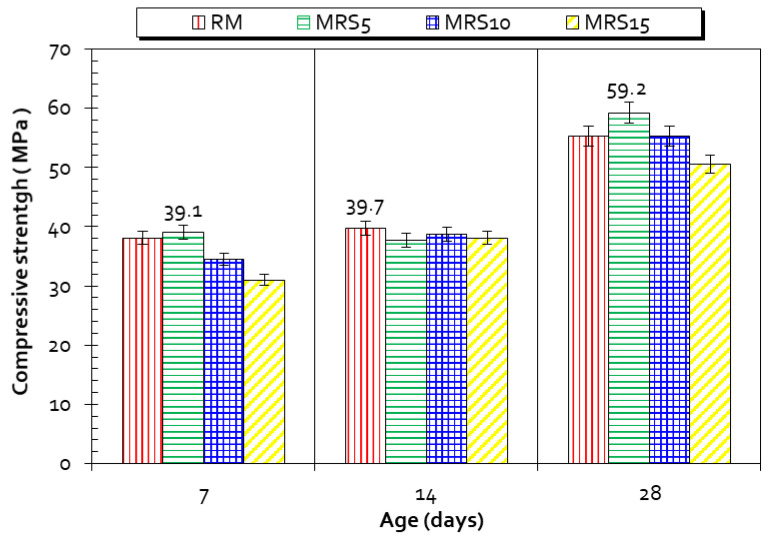
Compressive strength of RM and MRS, (5%, 10%, and 15%) mortars.

**Figure 10:**
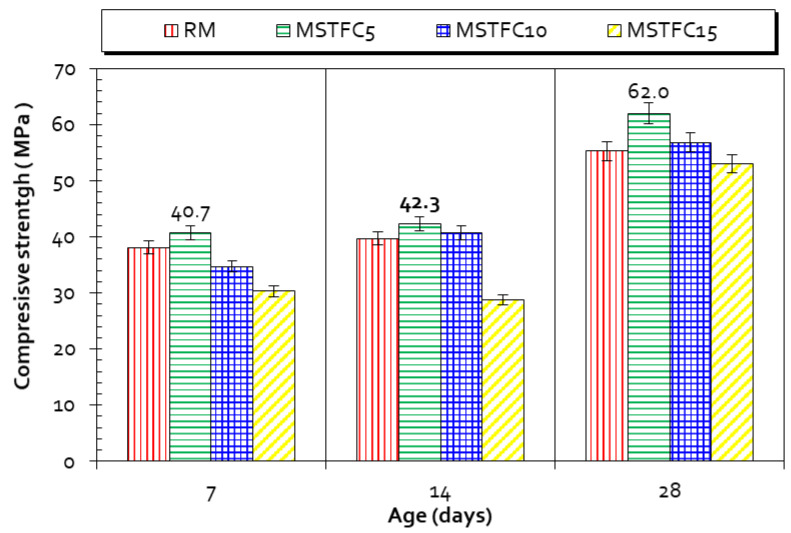
Compressive strength of RM and MSTFC (5%, 10%, and 15%) mortars.

The mechanical strength of prepared mortars was monitored following NF EN 196-1 [53]. Prismatic samples 4 × 4 × 16 cm^3^ were tested in compression for all formulated mortars. The results of the compression tests indicate that STCF-based mortars have significant resistance. After 28 days of maturation, the most resistant mortars were MSTFC5 and MRS5, with respective compressive strengths of 62.2 MPa and 59.2 MPa (Figure 9 and Figure 10). The substituted mortars containing 10% sediment exhibited the same strength as RM at 28 days, while the 15%-substituted mortars had lower strength than RM, in the range of 6% and 12%. The composition of mortars used to assess mechanical strength is given in Table 1. These results suggest that the presence of sediment, appropriately used in the cementitious matrix, results in an improvement in strength overall. Benezet et al. [77] demonstrated in a previous study that finely crushed particles of quartz (less than 5 µm or BET > 10,000 cm^2^/g) are highly active. In the present study, we must bear in mind that D50 ≤ 6 µm for all calcined material and BET > 20,000 cm^2^/g (Table 2). Therefore, one could argue that the flash calcination triggered the pozzolanic activity and impacted the materials’ fineness. This is correlated with the elimination of organic matter and densification.

**Figure 11:**
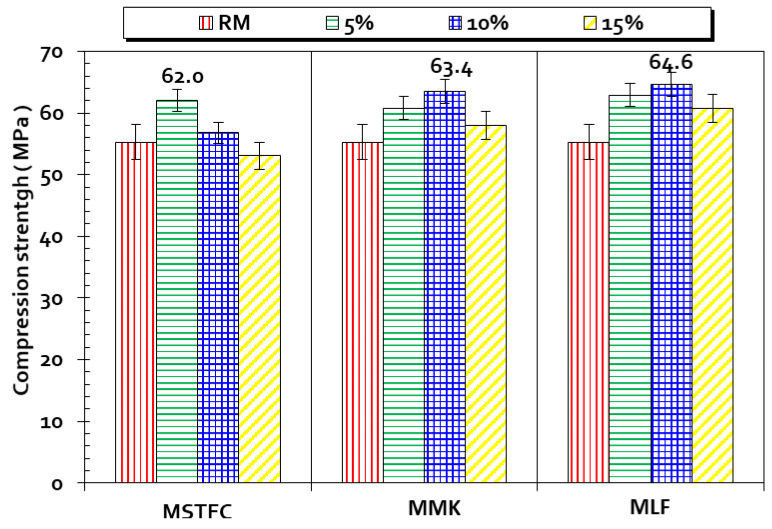
Compressive strength of MSTFC, MK and LF (5%, 10%, and 15%) mortars at 28 days maturation.

Finally, a comparison between RM, MRS, MSTFC, MK-based mortars, and LF-based mortars was performed (Figure 11), clearly showing that MSTFC5 mortars are similar to the MMK5 and MLF5 mortars after 28 days of maturation. Nonetheless, there is slightly less resistance for MSTFC10 and MSTFC15. Their resistance values are in the range of 13% to 15% less than those for mortars containing MK and LF. This can be explained by the fact that MK is a highly pozzolanic material. Its activity coefficient could be considered to be χ = (0.9 to 1) [30,78].

Studies conducted by Nicolas et al. [30] and Claverie et al. [29] on kaolin clays treated with flash calcination subsequently showed that the effect of the treatment should be recognized. It was demonstrated that physicochemical activity was noticed in the material after the clay phases had been activated. This suggests that calcined clays improve hydration reactions [2,26]. The decomposition of the physical and chemical effects indicates some strong connection between that physical effect and the phenomenon of heterogeneous nucleation. Nevertheless, the chemical effect seems to be controlled by complex chemical reactions, such as precipitation/dissolution, fineness [79], and amorphousness [26,80,81]. These effects probably triggered the enhancement observed in the mechanical performance [16,26,82,83,84]. Van Bunderen et al. [2,25] demonstrated that the addition of calcined dredging sediments reduces the autogenous shrinkage of cement and could potentially be used as an alternative SCM. According to Hentschel et al. [85] and Felekoglu [86], some factors such as grain shape and roughness can directly impact the rheology, fluidity, and porosity of mortars. The finely crushed minerals present can have some activity in the cementitious matrix [87].

### 3.8. Heat of Hydration—Langavant Calorimeter

**Figure 12:**
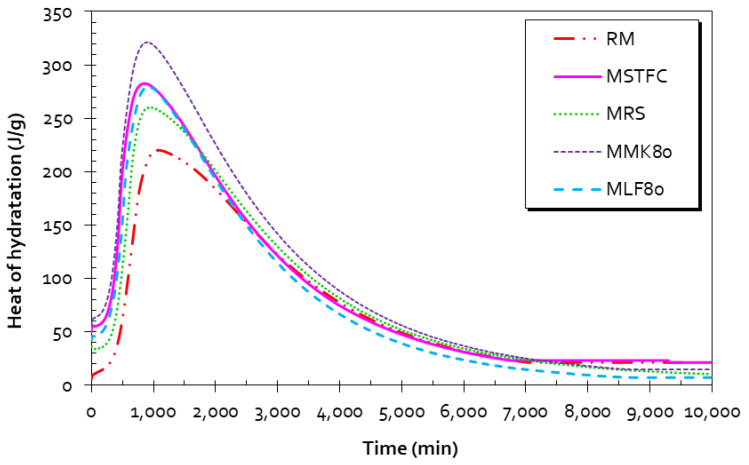
Relationship between the heat of hydration and time for RM, MRS, and MSTFC mortars.

The heat flow test can be considered a rapid screening test for supplementary cementitious materials (SCMs). The principle of this test is to monitor the variation in temperature and heat flow of mortars inside sealed measuring cells. The results are plotted in the graphs depicted in Figure 12. Mortar composition is reported in Table 6 (NF EN 196-9) [88], and the heat released can be deduced from the expression given in Equation (1). MK-based mortars are designated by MMK80, and MLF80 is an LF-based mortar. For the formulations, one substitution rate is tested (10%) because it seems to be the limit above which the compressive strength of MSTFC begins to decrease and becomes less than that of RM for a greater replacement rate.

(1) $$Q=\frac{C}{m_{c}}\theta_{t}+\frac{\int_{0}^{t}{\alpha *\theta }_{t}*\mathrm{dt}}{m_{c}}$$

where:

C = Total heat capacity (J/K);
$\theta_{t}$ = Temperature difference between the reference cell and measuring cell (K);
$m_{c}$ = Mass of cement (g);
α = Heat dissipation coefficient (J/(g*K);
t = Time (s).

RM reached a maximal temperature of 51.9 °C after 17.80 h, which corresponds to a generated heat of Q = 240.08 J/g. For MSTFC mortar, the heat released at the end of 15.90 h of screening was Q = 285.63 J/g, and MRS had a lower value. MMK80 is the most active mortar due to the high pozzolanic activity of MK (Q = 324.5 J/g). An increase in the heat flow was noticed following the replacement ratios of cement by SCM. This is mainly attributed to the filler effect (physical effect) but also partly to the early pozzolanic reaction (chemical effect) of sediments, as discussed by Snellings et al. [26].

The cumulative heat patterns demonstrate that MRS and MSTFC show higher heat than RM and appear to react faster. The finding is that additional thermic or chemical activity should be generated by the activated sediment particles. It can also be related to the lime phase, which reacts with water following Equation (2), or the hydration of hemihydrate (CaSO_4_·0.5H_2_O) in Equation (3) that produces additional heat output [89].

(2) $$\mathrm{CaO}+H_{2}O\;\to \;\mathrm{Ca}{\left(\mathrm{OH}\right)}_{2}+\;\mathrm{Heat}$$

(3) $$\langle {\mathrm{CaSO}}_{4}\;|0.5H_{2}O\rangle +1.5H_{2}O\;\to {\;\langle \mathrm{CaSO}}_{4\;}|2H_{2}O\rangle +\;\mathrm{Heat}$$

The cumulative heat patterns indicate that both MRS and MSTFC show greater heat release than RM and appear to lead accelerate chemical reactions. The result is that additional thermic or chemical activity could have been generated by sediment particles, possibly related to the lime phase and pozzolanic reactions. One possibility to lower this effect if it is not desired could be to use cement with a low heat of hydration, such as Portland-limestone cement (PLC). Skibsted and Snellings [41] demonstrated that two mechanisms limit SCM reactions: water availability and space accessibility in the cementitious matrix. These parameters should therefore have a real influence if a higher replacement ratio of cement to SCM is considered. Snelson et al. [90] led a similar study using two pozzolanic materials: metakaolin and fly ash. They showed that MK generates an enhancement of the overall hydration and the heat release is higher due to the beginning of rapid pozzolanic reactions. In cementitious materials, pozzolanic reactions are controlled by the supply of Ca^2+^ ions, as discussed by Snelson [90]. This can explain the heat excess generated by the sediments due to their high water requirements (typically ≅ 50%) and calcium content (≅15%). This suggests that sediments change heat release [23,26] and can efficiently enhance hydration reactions [25,26,30].

### 3.9. Initial Setting Time

The initial setting time test (VICAT) is the determination of the time it takes for the standardized needle to sink into the mixture to a depth of 6 mm. The results and mortar formulations are displayed in Table 7. RM is the mortar with the most rapid initial setting time (285 min), and the mortar with the longest time is the MRS (321 min). The MSTFC showed an initial setting time of 326 min.

**Figure 13:**
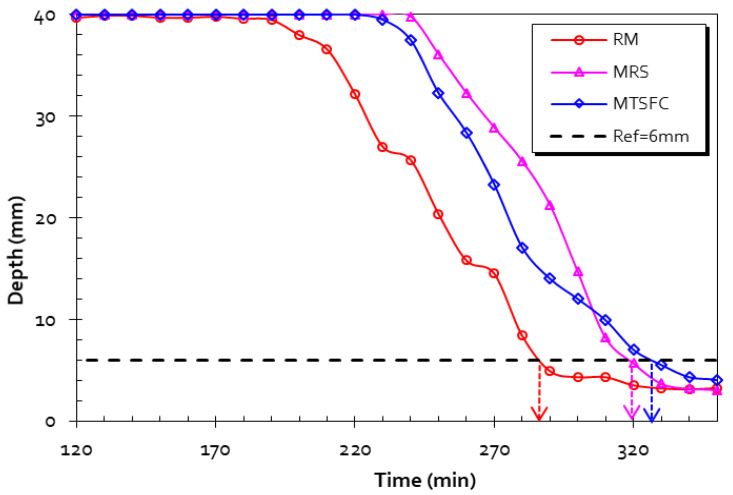
Evolution of the initial setting time for RM, MRS, and MSTFC.

Figure 13 shows the initial setting time test results. These patterns show that the initial setting time increases with the presence of sediment in the mortar formulation. At the beginning of the test, needle penetration was quite similar in the MRS and the MSTFC. We then argued that the presence of clayey phases or calcite in the sediments could explain this phenomenon by a contraction effect in the matrix. Hence, it can be concluded that there is no improvement in the setting process when sediments are used.

The initial setting time test (VICAT) results highlighted the fact that the presence of sediments leads to a delay in setting processes. This could be related to the presence of sulfates or contaminants (mineral or organic). Indeed, according to Arliguie and Grandet [72], the presence of zinc (Zn) has the most detrimental impact. Its presence leads strongly to lower compressive strength and decreases density. Zinc inhibits the hydration of C_3_S and C_3_A with a more pronounced effect on C_3_S. This phenomenon is due to the formation of an amorphous impermeable film around anhydrous grains, called zinc hydroxide (Zn(OH)_2_) (Equation (4)).

(4) $${\mathrm{Zn}}^{2+}+2{\left(\mathrm{OH}\right)}^{-}\to \;\mathrm{Zn}{\left(\mathrm{OH}\right)}_{2}$$

However, the presence of Ca^2+^ and OH^−^ ions seems likely to allow the destruction of the seal by the formation of a calcium hydroxyzincate layer and therefore enables hydration reactions to continue (Equation (5)).

(5) $$\mathrm{Zn}{\left(\mathrm{OH}\right)}_{4}^{2-}+{\;\mathrm{Ca}}^{2+}+2H_{2}O\;\to \;\langle \mathrm{CaZn}{\left(\mathrm{OH}\right)}_{4}|\;2H_{2}O\rangle$$

Similarly, the presence of nitrate (Ni) leads to a reduction in the hydration of cement by more than 50% [73,74]. Hemihydrate identified in XRD analysis (Equation (3)) leads into the production of gypsum (CaSO_4_, 2H_2_O), which should have a retarder effect in a cementitious matrix.

The initial setting time delay could be related to the presence of mineral or organic contaminants. Likewise, the marine nature of sediment probably accounts for this behavior. In fact, the presence of marine salt such NaCl, KCl, and sulphates could have caused a delay in the setting process, as demonstrated by Minocha et al. [73]. The presence of organic matter may also influence the hydration process. It was identified in a previous study to affect setting and hardening due to the formation of a particular protective film around the cement grains [91,92].

## 4. Conclusions

The effect of heat treatment processes on sediment performances was investigated in this paper. The purpose was to determine and compare the characteristics of flash-calcined sediment and regular SCMs. Sediment-based mortars were formulated and tested after 7, 14, and 28 days. The main conclusions that can be drawn from this work are the following:

Heat treatment modifies sediment properties such as density (by removal of organic matter), densification, and dehydroxylation. However, fineness is lowered due to a sintering/melting process.

XRD analysis highlights the fact that after flash calcination, the clayey phases are transformed mainly into illite. In addition, SEM analysis identified spherical particles made up of gases and aluminum silicates that could enhance the hydration process.

From a mechanical analysis, the presence of finely ground and flash-treated sediments improves mechanical resistance. The compressive strength of MSTFC mortars was 11.3% higher than that of the RM (control) at 28 days for 5% cement substitution, whereas the mechanical performances of MSTFC10 were equivalent to those of RM at 28 days.

The investigation conducted on the heat of hydration using the Langavant calorimetry method (NF EN 196-9) demonstrates that the presence of sediment generally modifies hydration kinetics and processes. It also shows that the presence of sediment generates additional heat release.

A later study of the durability and environmental impact of sediment-based mortars will be carried out in the near future.

## Figures and Tables

**Table 1:** Mortar composition of RM, MRS, MSTDC, and MSTFC for the heat of hydration test (NF EN 196-9) [50].

Constituent	RM	MRS			MSTFC			MMK80			MLF80		
Cement CEM I 52.5 N (g)—5%|10%|15%	450.0	427.5|405|382.5											
Sediment (g)	---	5%	10%	15%	5%	10%	15%	---			---		
		28.6	57.2	85.8	26.8	53.5	80.3						
Metakaolin (g)	---	---			---			5%	10%	15%	---		
								25.9	51.7	77.6			
Limestone filler (g)	---	---			---			---			5%	10%	15%
											26.2	52.5	78.7
Normalized sand (g)	1350	1350			1350			1350			1350		
Water (g)	225	225			225			225			225		

**Table 2:** Determination of the physical properties of the materials.

Materials	Cement (CEM I 52.5 N)	Raw Sediment (RS)	Flash Calcined Sediment (STFC)	Metakaolin (MK80)	Limestone Filler (LF80)	Natural Sand (NS)
Density (g/cm^3^)	3.15	2.48	2.65	2.74	2.70	2.65
Blaine (cm^2^/g)	3669	10,093	4106	4820	7181	4548
BET (cm^2^/g)	9194	86,207	59,930	94,600	9744	9507
TOC * (%)	---	5.76	0.99	---	---	---
LOI * (%)	1.90	9.92	1.70	---	---	---

TOC * = total organic carbon = 1.72 * LOI [61]; LOI * = loss of ignition.

**Table 3:** Concentrations (%) of the major oxide elements for OPC, RS, and STFC.

	SiO_2_	Al_2_O_3_	Fe_2_O_3_	CaO	SO_3_	K_2_O	TiO_2_	Na_2_O	MgO	P_2_O_5_	MnO	ZnO
OPC	20.0	5.1	3.4	63.5	3.1	0.8	0.3	0.3	0.8	0.4	0.1	0.2
RS	51.9	8.2	9.3	22.1	0.2	1.9	0.4	2.0	2.0	0.4	0.2	0.1
STFC	52.8	8.0	9.3	21.6	0.2	1.8	0.4	1.9	2.0	0.4	0.2	0.1

**Table 4:** Chemical composition of cement (%).

	Alite (C_3_S)	Belite (C_2_S)	Aluminate (C_3_A)	Ferrite (C_4_AF)	Anhydrite (CaSO_4_)	Gypsum (CaSO_4_, 2H_2_O)	Lime (CaO)
OPC	63.7	16.1	4.3	9.8	0.5	3.8	1.8

**Table 5:** Identification of clay minerals by the XRD method.

Samples	Smectite	Illite	Kaolinite	Chlorite	Interlayered 10- 14S Swellings	Additional Minerals
**RS**	32%	36%	20%	12%	---	Quartz
**STFC**	---	86%	5%	---	9%	Quartz

**Table 6:** Mortar composition of RM, MRS, and MSTFC for the heat of hydration test (NF EN 196-9) [50].

Constituent	RM	MRS	MSTFC	MMK80	MLF80
Cement CEM I 52.5 N (g)	360.0	331.8	329.8	328.7	329.2
SCMs (g)	---	28.2	30.2	31.3	30.8
Normalized sand (g)	1080.0	1080.0	1080.0	1080.0	1080.0
Water (g)	180.0	180.0	180.0	180.0	180.0

**Table 7:** Mortar composition of RM, MRS, MSTDC, and MSTFC for the initial setting time VICAT test (NF EN 196-3) [88].

Constituent	RM	MRS	MSTFC
Cement CEM I 52.5 N (g)	500.0	425.0	425.0
Calcined sediment (g)	0	75.0	75.0
Water (g)	150.0	165.0	165.0
Setting time	285 min (4 h:45 min)	321 min (5 h:21 min)	326 min (5 h:26 min)

## Data Availability

The data used in this study are available from the corresponding author on submission of a reasonable request.
